# hext, a software supporting tree‐based screens for hybrid taxa in multilocus data sets, and an evaluation of the homoplasy excess test

**DOI:** 10.1111/2041-210X.12490

**Published:** 2015-11-11

**Authors:** Kevin Schneider, Stephan Koblmüller, Kristina M. Sefc

**Affiliations:** ^1^ Institute of ZoologyUniversity of GrazUniversitätsplatz 28010GrazAustria; ^2^ Department of Systematic Botany and GeobotanyInstitute of Plant SciencesUniversity of GrazHolteigasse 68010GrazAustria

**Keywords:** AFLP, bootstrap support, Canidae, hybridization, homoplasy excess test, phylogenetics, SNP, *Vitis champinii*

## Abstract

1. The homoplasy excess test (HET) is a tree‐based screen for hybrid taxa in multilocus nuclear phylogenies. Homoplasy between a hybrid taxon and the clades containing the parental taxa reduces bootstrap support in the tree. The HET is based on the expectation that excluding the hybrid taxon from the data set increases the bootstrap support for the parental clades, whereas excluding non‐hybrid taxa has little effect on statistical node support. To carry out a HET, bootstrap trees are calculated with taxon‐jackknife data sets, that is excluding one taxon (species, population) at a time. Excess increase in bootstrap support for certain nodes upon exclusion of a particular taxon indicates the hybrid (the excluded taxon) and its parents (the clades with increased support).
2. We introduce a new software program, hext, which generates the taxon‐jackknife data sets, runs the bootstrap tree calculations, and identifies excess bootstrap increases as outlier values in boxplot graphs. hext is written in r language and accepts binary data (0/1; e.g. AFLP) as well as co‐dominant SNP and genotype data.
3. We demonstrate the usefulness of hext in large SNP data sets containing putative hybrids and their parents. For instance, using published data of the genus *Vitis* (˜6,000 SNP loci), hext output supports *V. *×* champinii* as a hybrid between *V. rupestris* and *V. mustangensis*.
4. With simulated SNP and AFLP data sets, excess increases in bootstrap support were not always connected with the hybrid taxon (false positives), whereas the expected bootstrap signal failed to appear on several occasions (false negatives). Potential causes for both types of spurious results are discussed.
5. With both empirical and simulated data sets, the taxon‐jackknife output generated by hext provided additional signatures of hybrid taxa, including changes in tree topology across trees, consistent effects of exclusions of the hybrid and the parent taxa, and moderate (rather than excessive) increases in bootstrap support. hext significantly facilitates the taxon‐jackknife approach to hybrid taxon detection, even though the simple test for excess bootstrap increase may not reliably identify hybrid taxa in all applications.

## Introduction

**Figure 1:**
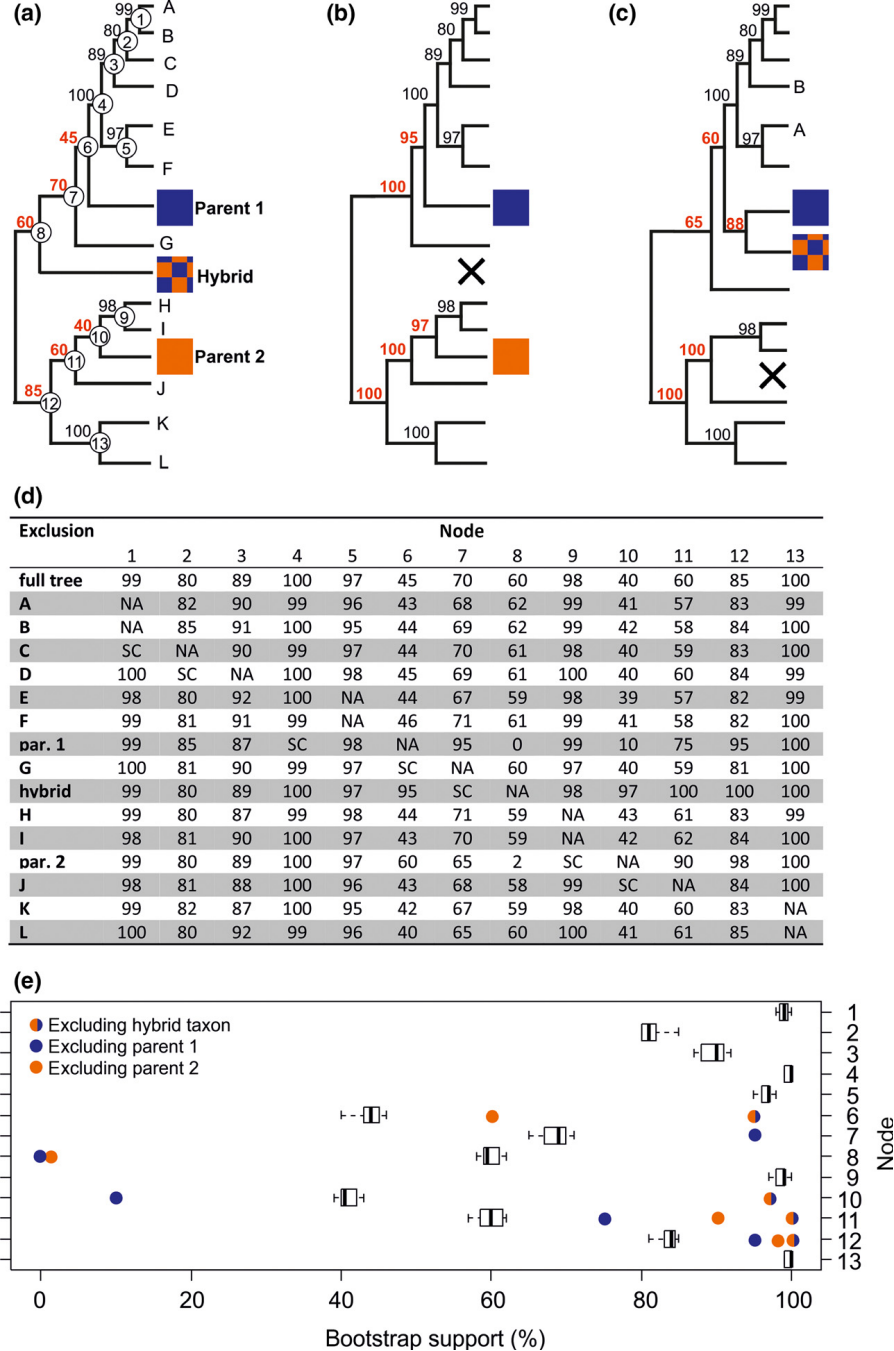
Excess homoplasy introduced by a hybrid taxon in a multilocus phylogenetic tree. (a) The hybrid is placed intermediate to the parental taxa. Bootstrap support (numbers above branches) for clades containing the parental taxa is low due to homoplasy with the hybrid. Circled numbers identify nodes. (b) Exclusion of the hybrid increases bootstrap support for clades containing the parental taxa. (c) Exclusion of one parent taxon causes changes in BS support and tree topology: increased bootstrap support for both parental clades, and placement of the hybrid with its other parent. (d) BS values for each node observed in the full tree. BS values in the full tree (first line) and in each taxon‐jackknife tree are compiled in table. SC, support carryover: BS values were not scored for nodes that were sister to the excluded taxon. NA, node had joined the excluded taxon. (e) Boxplots representing the distribution of BS values scored in the taxon‐jackknife trees for each node observed in the full tree. The boxes encompass 50% of the observed values that are located between the first and the third quartile and define the interquartile range (IQR). Vertical bars within boxes mark the median value. Whiskers extend to the smallest and the largest BS value located within the 1·5 × IQR distance from the boxes, whereas values beyond this distance are considered outliers and represented by dots. Dot colour indicates whether an outlier was caused by the exclusion of the hybrid, parent 1 or parent 2.

Hybridization has contributed significantly to the generation of biological diversity (Abbott & Rieseberg 2012), and efficient means to screen for hybrid taxa are needed to improve our understanding of its importance across taxonomic groups. The homoplasy excess test (HET) proposed by Seehausen (2004) is an intuitive approach to the detection of hybrid taxa in multilocus nuclear trees. The method relies on the fact that the multilocus genotypes of hybrid taxa are mosaics of the hybrid parents' alleles. Across loci, hybrid taxa will appear intermediate to the parent taxa, but individual alleles will be identical with one or the other hybrid parent. This introduces homoplasy between the hybrid taxon and the clades containing the parental taxa and reduces bootstrap support in the tree (Fig. 1a). Removing the hybrid taxon from the data set should consequently increase bootstrap support for clades that include the parental taxa or their descendants (Fig. 1b), and exclusion of a parent taxon should increase the support for the clade in which it was previously placed (Fig. 1c). Excluding non‐hybrid and non‐parent taxa, in contrast, should have only small effects. To carry out a HET, taxon‐jackknife data sets are generated by excluding one taxon (species, population) at a time. Bootstrap (BS) trees are calculated with the full and taxon‐jackknife data sets, and BS values for all nodes of the full tree are recorded (Fig. 1d). The distributions of BS values for each of the nodes are then visualized in boxplots, and outliers are identified, for example in boxplot analyses (Fig. 1e). Boxplot outliers point out potential hybrid and parent taxa (taxa whose exclusion generated the outlier BS value) and the phylogenetic position of the parent taxa (within the node that received the outlier BS value).

Hence, on theoretical grounds, the HET promises to be an efficient exploratory tool to screen multilocus phylogenetic data sets for candidate hybrid taxa, which can then be further analysed, for example by coalescence model‐based analyses or population genetic approaches. It can also be used to investigate whether suspected hybrid taxa produce the expected signal. So far, the test has mainly been applied to AFLP data, where the results suggested an important role of hybridization in the diversification of cichlid fish, sailfin silversides, and clownfish as well as hybrid speciation in fruit‐eating Caribbean bats (Table 1). However, there have been no investigations on the test's likelihood to produce false‐positive or false‐negative results.

**Table 1:** Examples of HETs used to screen multilocus phylogenies for hybrid taxa

Taxon group	Reported upper outliers/examined nodes (if reported)^a^	Polymorphic markers used in HET	Tree size (number of individuals, taxa)	Number of exclusion experiments (exclusion sets^b^)	Outlier detection method	Genetic distance; tree‐building algorithm	References
Cichlid fish (Cichlidae)							
Cameroon crater lake cichlids^c^	10/30 nodes	(a) 2355 AFLP (b) 530 AFLP	(a) *n* = 32, 16 taxa (b) *n* = 80, 14 taxa	(a) 16 (taxa) (b) 14 (taxa)	Boxplot, >1·5 IQR	Link *et al*. (1995); NJ	Schliewen & Klee (2004)
Austrotilapiini	2/selected focus nodes	5 nuclear gene sequences	*n* = 63, 54 species	63 (individuals)	Boxplot, >1·5 IQR	GTR+3; ML	Schwarzer *et al*. (2009)
*Steatocranus*	54/48 nodes	1706 AFLP	*n* = 141	45 (clades)	Boxplot, >1·5 IQR	Link *et al*. (1995); NJ	Schwarzer *et al*. (2012b), Schwarzer, Misof & Schliewen (2012a)
Haplochromini	61/60 nodes	1984 AFLP	*n* = 68, 48 species	86 (species and clades)	Boxplot, >1·5 IQR	Link *et al*. (1995); NJ	Schwarzer *et al*. (2012b)
*Amphilophus*	5	1351 AFLP	*n* = 102	102 (individuals)	Boxplot, >1·5 IQR	Link *et al*. (1995); NJ	Geiger, McCrary & Schliewen (2010)
Bower‐building Lake Malawi cichlids	1	3171 AFLP	*n* = 60, 19 species	19 (species)	Boxplot, reported outlier >3 IQR	Nei & Li (1979); NJ	Kidd, Kidd & Kocher (2006)
Tropheini	6/selected focus nodes	1258 AFLP	*n* = 104, 26 taxa (species and undescribed taxa)	21 (species and undescribed taxa)	Boxplot, >3 IQR	Nei & Li (1979); NJ	Koblmüller *et al*. (2010)
*Tropheus*	14 reported (selected examples)/15 nodes	768 AFLP	*n* = 117, 51 populations	53 (populations and combinations of populations)	Boxplot, >3 IQR	Nei & Li (1979); NJ	Egger *et al*. (2007)
East African cichlids	34	3282 AFLP	*n* = 94, 91 species	414 (species, clades, random sets)	Boxplot, >3 IQR	Jaccard, NJ	Weiss, Cotterill & Schliewen (2015)
*Xenotilapia*	0	2478 AFLP	*n* = 32, 11 species	11 (species)	Boxplot, >1·5 IQR	Nei & Li (1979); NJ	Kidd *et al*. (2012)
Limnochromini	0/8 nodes	1128 AFLP	*n* = 31, 9 species	9 (species)	Boxplot, >1·5 IQR	Nei & Li (1979); NJ	P. C. Kirchberger & S. Koblmüller unpublished
*Bathybates*	0/6 nodes	659 AFLP	*n* = 38, 7 species	7 (species)	Boxplot, >1·5 IQR	Nei & Li (1979); NJ	Kirchberger *et al*. (2012)
Sailfin silversides, Telmatherinidae	1/1 node	1327 AFLP	*n* = 74, 6 taxa	>100 (taxa and random sets)	histogram	Link *et al*. 1995; NJ	Herder *et al*. (2006)
Fruit‐eating bats, Phyllostomidae	2/6 nodes	374 AFLP	*n* = 73, 8 species	8 (species)	Boxplot, reported outliers ≫3 IQR	Nei & Li 1979; NJ	Larsen, Marchán‐Rivadeneira & Baker (2010)
Clownfish, Pomacentridae	13/40 nodes	7 nuclear gene sequences	*n* = 41, 27 species	>27 (27 species plus combinations of species)	Boxplot, >1·5 IQR	GTR+G; ML	Litsios & Salamin (2014)

a Studies often report outliers only for selected nodes.

b Exclusion experiments were conducted by excluding one taxon at a time (e.g. Individual/species/population/clade), by excluding multiple taxa at a time, or by excluding random sets of individuals.

c This study combined the results of two HETs with data sets (a) and (b).

Lacking software to automate the HET procedure, the numerous input and output files have been generated and parsed manually in previous work. The tediousness of this procedure and the necessity to evaluate the test prompted us to design a new software program, hext, which runs all steps automatically and compiles the output ready for interpretation. We confirm the functionality of the program by replicating published HETs, demonstrate its applicability to large empirical SNP data sets, and evaluate the performance of the taxon‐jackknifing approach with simulated SNP and AFLP data.

## Methods

### Overview of hext

#### Computation of phylogenetic trees

Input handled by hext includes binary data (e.g. AFLP or RFLP coded as 1/0 for presence/absence of a band), SNP data represented by nucleotide states (AG GG TC AA, etc.) allowing >2 alleles per site, and bi‐allelic markers represented by genotype codes 0 (AA), 1 (AB) and 2 (BB). Missing data are allowed. The use of an outgroup is optional. The number of ingroup taxa must be large enough to allow outlier detection among the BS values generated by taxon‐jackknifing. In previous applications of the test, data sets comprised 7 to >90 taxa (Table 1).

hext is written in r language (R Development Core Team 2014) and partly relies on functions of the ape (Paradis, Claude & Strimmer 2004; Popescu, Huber & Paradis 2012) and phangorn packages (Schliep 2011) for phylogenetic analyses. Parallel computing is achieved via the r packages ‘foreach’ and ‘domc’ on unix‐based operating systems (linux, mac os x) or ‘dosnow’ on Windows. A full list of hext functions is given in Appendix S1.

Nei & Li (1979) and allele‐sharing distances are calculated for binary and SNP data, respectively (function ‘seq_dist’). Trees are estimated by neighbour‐joining (Saitou & Nei 1987; ape function ‘nj’) to keep computation times down. As node support is evaluated by bootstrapping across loci (Felsenstein 1985; ‘seq_dist’), the total number of trees calculated in the course of a HET run easily amounts to several tens of thousands (number of taxon‐jackknives times number of bootstrap replicates).

hext starts with the calculation of the phylogenetic tree (and BS values) for the total set of taxa (the ‘full tree’), and repeats the analysis for each of the desired taxon‐jackknife data sets (‘taxon‐jackknife trees’; function ‘get_trees’). Groups of samples to be excluded in the jackknifing procedure are either listed in a separate file or specified in the genotype data file by the addition of group identifiers to the sample names.

#### Evaluation of bootstrap support values

BS values for all nodes in the full and taxon‐jackknife trees are recorded. hext calls the functions prop.part and prop.clades implemented in ‘ape’ to list all bipartitions occurring in the bootstrap tree series calculated with each data set (full and taxon‐jackknife) and to determine the number of times they occur in each tree series. BS values pertaining to the nodes present in the full tree are then retrieved from these data (functions ‘node_comp’, ‘parse_trees’) and reported in text and graphical output (‘store_plot’). Furthermore, BS values for nodes not occurring in the full tree but in one or more taxon‐jackknife trees are also saved (‘alttop_search’) and are of interest when the monophyly of a taxon is disrupted due to the affinity between hybrid and parent in the tree (see *Vitis* example below). The program offers options to access these data (‘access_custom’).

Excess homoplasy is inferred when the exclusion of a certain taxon increases the BS value for a certain node much more than does the exclusion of other taxa. Following previous applications of the HET (Table 1), BS outliers are diagnosed in boxplots. The outlier criterion can be set by the user as *x* times the interquartile range (IQR) below and above the first and third quartile, respectively; previous applications followed Tukey (1977) using *x *=* *1·5 and *x *=* *3 (Table 1). Lower outliers (excessive reductions of BS support of a node) can be informative by identifying clades that are due to the affinity between hybrids and their parents and therefore disappear upon exclusion of the hybrid taxon. Examples are given below in the analysis of the *Vitis* and Canidae data. hext outputs BS values for each node in each taxon‐jackknife tree in a table, which can be used as input for alternative outlier tests (e.g. Vidmar & Blagus 2014).

#### Support carryover

Removing a taxon from a phylogenetic tree will in most cases increase the bootstrap support for its sister clade irrespective of hybridization‐induced homoplasy. For example, the removal of taxon C from the tree in Fig. 1a would increase BS support for the sister clade (A,B), because the characters that joined taxon C to its sister clade now add support to this node, a phenomenon called ‘support carryover’ by Weiss, Cotterill & Schliewen (2015). hext accounts for it (functions ‘sisternodes’, ‘boot_excl’) and ignores BS values of clades that are sister to the excluded taxon in the full tree (replaced by ‘SC’ in Fig. 1d).

#### Output and further analysis options

**Figure 2:**
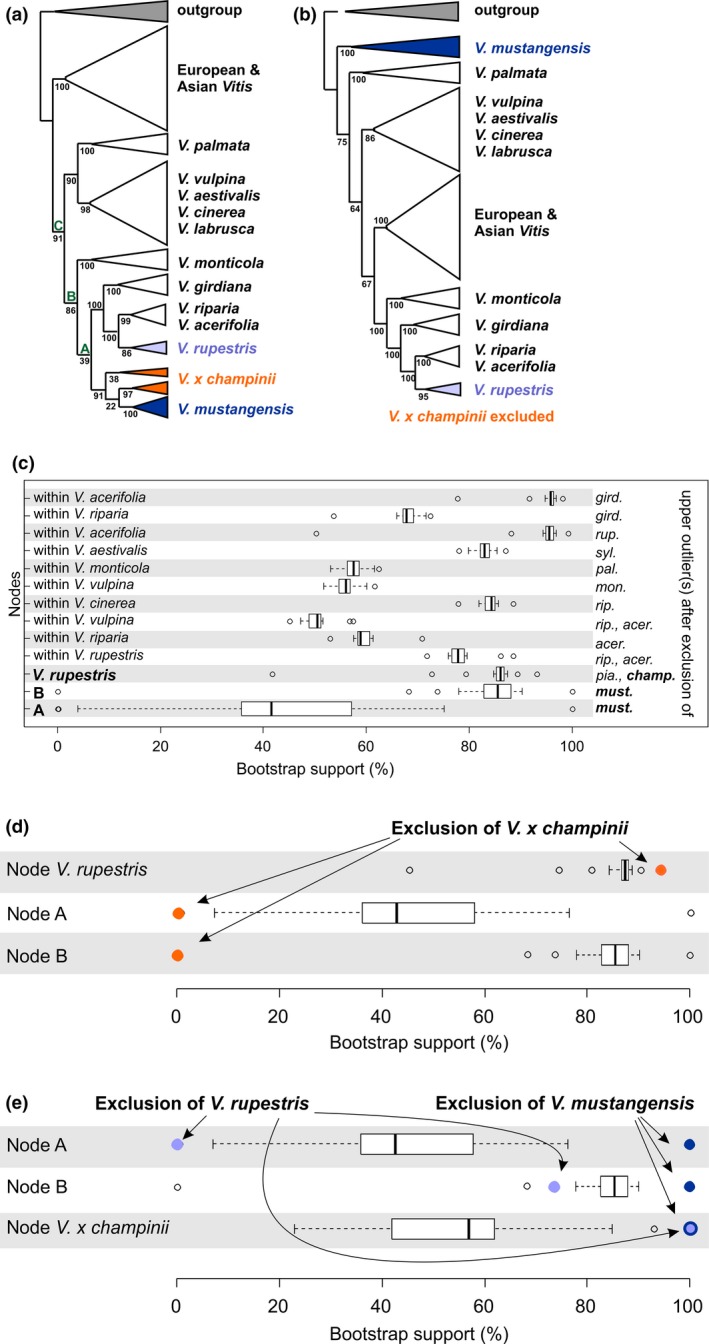
Homoplasy excess test results for the *Vitis* SNP data set. *V. *×* champinii* is a natural hybrid between *V. rupestris* and *V. mustangensis*. Trees were rooted with *V. rotundifolia*, and nodes are labelled with BS values. (a) The full tree, containing hybrid and parent taxa. (b) Tree obtained after exclusion of the hybrid, *V. *x* champinii*. (c) Graphical output of hext, showing boxplots of BS values across taxon‐jackknife trees for nodes at which at least one outlier value was detected at a distance >1·5 IQR from the box. Description of the nodes (on left vertical axis) and identification of species whose exclusion caused the upper BS outlier(s) were added manually. Bold font highlights nodes and taxa contributing the expected hybrid signals. Nodes A and B are identified in 2a, node “*V. rupestris*” joins the five *V. rupestris* accessions. *gird*., *V*.* girdiana*;* rup*., *V*.* rupestris*;* syl*., *V*.* sylvestris*;* pal*., *V*.* palmata*;* mon*., *V*.* monticola*;* rip*., *V*.* riparia; acer*., *V*.* acerifolia*;* pia*,* V. piasezkii*;* champ*., *V*.* *× *champinii*;* must*., *V*.* mustangensis*. (d, e) Annotated boxplots revealing reduced homoplasy and tree topology changes after exclusion of the putative hybrid taxon *V. *×* champinii* (d) and its parental species *V. rupestris* and *V. mustangensis* (e). The node defining monophyly of *V. *×* champinii* did not occur in the full tree (2a) and is therefore not included in the default hext output shown in 2c. The boxplot for this node was obtained using the hext option to create custom boxplots for defined nodes. Both exclusion of *V. rupestris* and of *V. mustangensis* yielded a BS of 100% (overlapping signals drawn as light‐blue circle with dark‐blue ring). The smaller of the two upper outliers at the *V. *×* champinii* node originates from the tree excluding *V. acerifolia*, and is not interpreted as evidence of the putative hybrid origin of *V. *×* champinii*. Similarly, in (d), the smaller of the two upper outliers at the *V. rupestris* node originates from the tree excluding *V. piasezkii*, and is also not interpreted as evidence of the putative hybrid origin of *V. *×* champinii*. Other upper outliers not highlighted in (d) are due to the exclusion of a parent as annotated in (e). Lower outliers highlighted in neither (d) nor (e) are also not considered connected to the putative hybrid origin of *V. *×* champinii*.

hext output includes newick tree files with information on the full and all taxon‐jackknife trees, a tabular compilation of BS values calculated with the full and taxon‐jackknife data, tabular information on BS outliers, and boxplot graphs in PDF and EPS formats illustrating the distribution of BS values for each node in the full tree. Additionally, the output includes BS values for all nodes that are found in any of the bootstrap analyses of full and taxon‐jackknife data sets (including, for instance, nodes occurring only in a jackknife tree but not in the full tree, and nodes which are only present in trees constructed in the process of bootstrapping). Furthermore, hext offers options to create custom boxplots for selected nodes and to retrieve information on nodes which do not occur in the BS tree of the full taxon set (e.g. the node joining all *V. *×* champinii* in the *Vitis* example, Fig. 2; functions ‘access_custom’, ‘access_alttop’).

#### Functionality of the program

The functionality of hext was tested with three AFLP data sets, for which homoplasy excess tests had already been carried out by running BS analyses in PAUP (Swofford 2003) and compiling the BS values manually (Egger *et al*. 2007; Koblmüller *et al*. 2010; S. Koblmüller & P. C. Kirchberger, unpublished data). In all cases, hext produced the same results as the manual analyses.

#### Computation times

Computation times varied with the type of data, the size of the data set as well as the number of requested jackknife trees and bootstrap replicates. For instance, analysis of the large canid data set (47 845 loci, data type: SNP genotype, 142 individuals, 18 jackknife sets, 1000 BS replicates) took 2 day 4 h 58 min; analysis of a simulated SNP data set (1279 loci, data type: SNP nucleotide, 40 individuals, 48 jackknife sets, 1000 BS replicates) took 6 h 54 min, both running two parallel threads on a DELL OPTIPLEX 3020, 64‐bit, 8 GB RAM, Intel(R) Core(TM) i5‐4570 CPU @ 3·20 GHz (4 threads) & 6 MB cache, Windows 7. Analysis of a simulated AFLP data set (5003 loci, data type: binary, 40 individuals, 19 jackknife sets, 1000 BS replicates) running 4 parallel threads on a DELL OPTIPLEX 9020, 64‐bit, 16 GB RAM, Intel(R) Core(TM) i7‐4770 CPU @ 3·40 GHz (8 threads) & 8 MB cache, Windows 7 Professional took only 21 min.

### Simulations of SNP and AFLP Data Sets to Evaluate the Performance of the HET

MCcoal (part of bp&p version 3.1) was used to simulate SNP and AFLP polymorphism along a predefined tree, assuming free recombination between loci (Rannala & Yang 2003). Numbers of simulated loci ranged from several tens to several hundreds of thousands, and resulted in data sets ranging from ~300 to ~5000 polymorphic loci (Table 2).

**Table 2:** Homoplasy excess tests (HETs) with simulated data sets. Except for #7, polymorphisms were simulated on the tree topology shown in Fig. 3a. Columns headed ‘simulated data sets’ provide information on simulations and resulting trees (number of polymorphic loci resulting from the simulation; θ, MCcoal population size parameter; hybrid τ, MCcoal divergence time parameter for the origin of the hybrid taxon; mean BS, average bootstrap values for nodes in the full tree). Columns headed ‘signals related to hybrid taxon’ report the number of nodes at which upper BS outliers or maximum BS values were observed upon exclusion of the hybrid taxon or of a taxon descending from one of the hybrid's parents (IQR, interquartile range). Columns headed ‘false‐positive upper boxplot outliers’ report the total number of BS outliers upon exclusion of non‐hybrid taxa. In the last column, only nodes joining at least two different taxa were considered

Simulated data sets					Signals related to hybrid taxon				False‐positive upper boxplot outliers		
					Exclusion of hybrid taxon			Exclusion of parent taxon			
No.	Marker loci	θ	Hybrid τ	Mean BS	Upper boxplot outliers >1·5 × IQR (indicated parent lineage)	Upper boxplot outliers >3 × IQR	Maximum BS, but not outlier, at >1·5 × IQR (indicated parent lineage)	Upper boxplot outliers >1·5 × IQR (excluded parent)	>1·5 × IQR	>3 × IQR	for nodes ≥2 taxa; >1·5 × IQR
No hybrid taxon											
1	362 SNP	0·00001	n.a.	80·48	n.a.	n.a.	n.a.	n.a.	0	0	0
2	987 AFLP	0·00001	n.a.	97·51	n.a.	n.a.	n.a.	n.a.	2	0	1
3	645 SNP	0·0005	n.a.	58·88	n.a.	n.a.	n.a.	n.a.	21	12	14
4	1310 SNP	0·0005	n.a.	73·25	n.a.	n.a.	n.a.	n.a.	13	8	7
5	4001 AFLP	0·0005	n.a.	80·48	n.a.	n.a.	n.a.	n.a.	9	4	4
6	4910 SNP	0·0005	n.a.	84·02	n.a.	n.a.	n.a.	n.a.	3	3	0
7	5293 AFLP (radiation)	0·0005	n.a.	65·95	n.a.	n.a.	n.a.	n.a.	10	6	10
Hybrid taxon: s × l											
8	1726 AFLP	0·0005	0·00001	63·25	1 (s)	0	2 (l)	0	18	11	10
9	1285 SNP	0·0005	0·00001	76·55	2 (s)	2	0	2 (s)	12	7	3
10	5002 AFLP	0·0005	0·00001	79·81	0	0	1 (s), 1 (l)	0	4	2	2
11	5088 SNP	0·0005	0·00001	89·26	1 (l)	0	4 (s)	1 (l)	5	1	0
12	5003 AFLP	0·0005	0·000002	79·52	0	0	3 (s), 2(l)	0	4	0	0
Hybrid taxon: (r,q) × k											
13	640 SNP	0·0005	0·000025	58·02	3 (r,q)	2	0	1 (r)	19	9	9
14	1328 AFLP	0·0005	0·000025	64·16	2 (r,q)	0	1 (r,q)	1 (q)	16	4	9
15	1284 SNP	0·0005	0·000025	70·07	2 (r,q)	1	0	1 (r)	12	6	2
16	5047 SNP	0·0005	0·000025	82·95	0	0	1 (r,q)	0	3	1	0
17	409 SNP	0·00001	0·000025	88·66	2 (r,q)	0	1 (r,q), 1 (k)	0	5	1	0
18	385 SNP	0·00001	0·000025	91·58	1 (k)	0	3 (r,q)	0	7	0	0
19	787 SNP	0·00001	0·000025	95·24	0	0	2 (r,q), 1 (k)	0	2	0	0
20	784 SNP	0·00001	0·000025	97·13	0	0	3 (r,q), 1 (k)	0	0	0	0
21	964 AFLP	0·00001	0·000025	98·28	1 (k)	1	2 (r,q)	0	5	0	0
Hybrid taxon: (r,q) × (l,m,n)											
22	1672 AFLP	0·0005	0·000037	63·91	0	0	1 (r,q)	2 (m); 1 (l)	18	5	7
23	1279 SNP	0·0005	0·000037	64·02	2 (r,q)	1	0	0	16	10	2
24	5160 SNP	0·0005	0·000037	85·20	0	0	1 (r,q)	0	4	0	0
25	375 SNP	0·00001	0·000037	90·37	0	0	3 (r,q)	0	2	0	0
26	400 SNP	0·00001	0·000037	91·02	0	0	1 (r,q)	0	4	3	1

**Figure 3:**
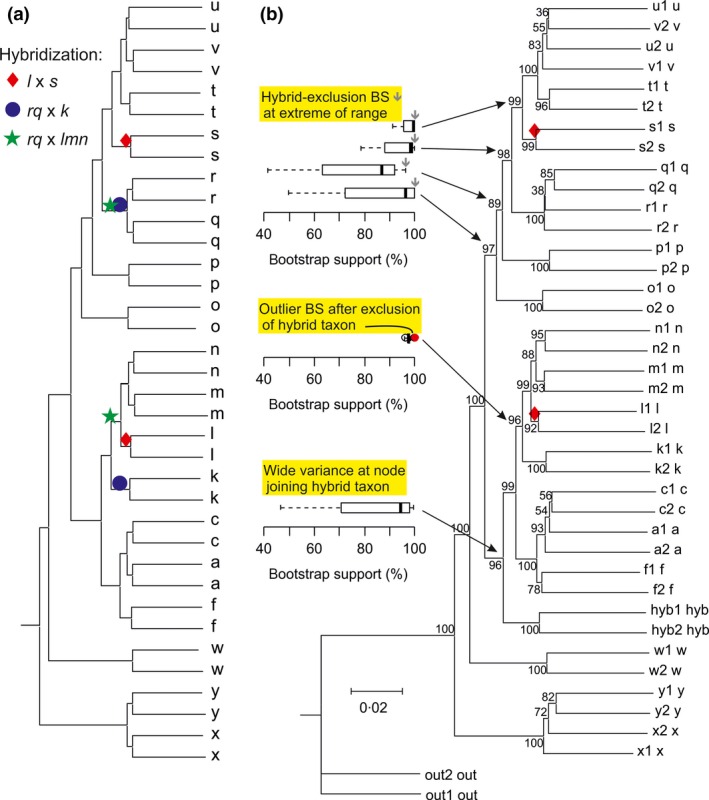
(a) Tree topology used to simulate AFLP and SNP data sets. Simulations assumed that hybridization between two lineages marked with corresponding symbols gave rise to a novel hybrid taxon. (b) Full tree obtained from the SNP data set in simulation #11 (Table 2) including a hybrid taxon originating from hybridization between the lineages marked with red diamonds (*l* × *s*). BS support is given near nodes (% in 1000 BS replicates). BS boxplots are shown for selected nodes to illustrate the patterns described in the text.

AFLP polymorphisms originated from point mutations in restriction sites (a total of 10 bp) and were translated into a binary 0/1 matrix accounting for dominance of the allele with two intact restriction sites. The tree topology (Fig. 3a) was based on the AFLP tree of the cichlid tribe Tropheini (Koblmüller *et al*. 2010) and contained 18 ingroup taxa, 1 outgroup as well as, when indicated, one hybrid taxon, with 2 individuals per taxon. Divergence time parameters τ, defined as average number of mutations per site, ranged from τ = 0·000225 for the MRCA of the ingroup to τ = 0·00001 for the most recent splits between sister taxa. Additionally, one data set was simulated as a radiation at τ = 0·00005–0·000058 with approximately equal distances among all 18 ingroup taxa, and two data sets included 36 ingroup taxa on a tree constructed by mirroring the ingroup topology shown in Fig. 3a. Different population size parameters (θ, defined as 4N_e_μ) were used to vary the rate of lineage sorting. Variable numbers of loci yielded further variation in tree support (Table 2).

To simulate a hybrid taxon, two separate MCcoal simulations were run, one with the hybrid taxon branching off from one parent and another one with the hybrid taxon branching off from the other parent. By merging the data sets resulting from these two simulations into one data set, the hybrid taxon's genotype was composed of unlinked markers inherited from the two parents. The divergence of the hybrid taxon from the parental taxa corresponds to the hybridization event that gave instant rise to the novel hybrid taxon. Both data sets were simulated with the same number of loci, such that the parent taxa contributed equally to the hybrid taxon. Different time parameters were set for the origin of hybrid taxa: τ = 0·000037 for the ancient hybridization between the lineage ancestral to node (r,q) and the lineage ancestral to the node (l,m,n); τ = 0·000025 for the hybridization between the lineage ancestral to node (r,q) and the lineage ancestral to taxon k; τ = 0·00001 and, in one simulation, τ = 0·000002 for the recent hybridization between the lineages ancestral to taxa l and s, respectively (Fig. 3a).

Homoplasy excess tests with simulated data sets were based on 1000 bootstrap replicates for the full and each taxon‐jackknife data set. Jackknife data sets were generated by excluding one taxon at a time. In addition, in some analyses, we additionally excluded randomly chosen individuals (29 random sets, each with two individuals of different taxa).

## Results and discussion

### Example Applications to Empirical Data

#### Hybrid signal in a SNP‐based phylogeny of the genus *Vitis* (grape and relatives)

The North American *Vitis *×* champinii* is considered a natural hybrid between *V. mustangensis* (syn. *V. candicans*) and *V. rupestris* (Division of Agriculture and Natural Resources, University of California, http://iv.ucdavis.edu/Viticultural_Information/?uid=166&ds=351). We tested the ability of hext to retrieve a hybrid signal for *V. *×* champinii* with the SNP data set generated by Miller *et al*. (2013). A total of 6114 SNP genotypes of 1173 accessions representing 18 *Vitis* species, including *V. *×* champinii* and its putative parent species, were derived from the Vitis9kSNP array. The HET does not require large sample sizes per species, and no more than five accessions per species (those with the least amount of missing data) were included in the analysis. A total of 1000 bootstrap replicates were calculated with the full and with each taxon‐jackknife data set. For taxon‐jackknifing, one species at a time was removed from the data set.

In the full tree, almost all species were monophyletic with 100% BS support (Fig. 2a). Exceptions were *V. sylvestris* (paraphyletic with respect to *V. vinifera*), *V. acerifolia* (paraphyletic with respect to *V. riparia*), *V. rupestris* (86% BS support), and the putative hybrid *V. *×* champinii*, which was paraphyletic with respect to one of its putative parents, *V. mustangensis* (Appendix S2). The HET identified a total of 16 upper BS outliers >1·5 × IQR at 13 different nodes (Fig. 2c). Ten of these nodes connected individuals of the same species, with outliers often caused by the exclusion of a closely related species. These are examples of outlier signals that would not be interpreted as indications of hybrid taxa. In contrast, three of the four outliers at the remaining three nodes as well as topology changes in the taxon‐jackknife trees supported a hybrid origin of *V. *×* champinii* from *V. mustangensis* and *V. rupestris*:

(1) Exclusion of *V. *×* champinii* from the data set increased BS support for *V. rupestris* from 86% to 95%, which was a considerably greater change in BS support than observed in the other taxon‐jackknife trees (Fig. 2d). Additionally, excluding *V. *×* champinii* released the other putative hybrid parent, *V. mustangensis*, from its weakly supported affiliation with the clade containing *V. rupestris* and placed it at a basal position as sister to the remaining ingroup (Fig. 2b, Appendix S2). Accordingly, BS support for nodes A and B, which joined *V. mustangensis* with the *V. rupestris* clade, dropped to zero (Fig. 2d). The position of *V. mustangensis* after exclusion of *V. *× *champinii* is in accordance with the placement of *V. mustangensis* in a basal lineage of the nuclear multigene tree by Wan *et al*. (2013), which also did not include *V. *×* champinii*.

The effect of the presence of *V. *×* champinii* on the placement of the putative hybrid parent *V. mustangensis* can be explained by the tug‐of‐war between a hybrid and its parents. The position of one putative hybrid parent, *V. rupestris*, is anchored by the presence of several closely related species in the phylogeny (Fig. 2a, Appendix S2), whereas species closely related to *V. mustangensis* (e.g. *V. shuttleworthii*,* V. nesbittiana*; Wan *et al*. 2013) are not included in the present data. *V. rupestris*, having a strong foothold within its clade, pulls its putative hybrid offspring *V. *×* champinii* towards itself, which in turn draws the other hybrid parent, *V. mustangensis*, away from its basal position towards a sister relationship with *V. *× *champinii*.

(2) Excluding the parent, *V. mustangensis*, increased BS values for nodes grouping the remaining North American *Vitis* species (nodes A, B, C in Fig. 2a), and produced upper outliers in the BS value distributions for nodes A and B (Fig. 2e). These BS increases are consistent with the hypothesis that *V. mustangensis* did not evolve within this clade, but was drawn into it by its hybrid offspring *V. *× *champinii*.

(3) Exclusion of the other parent, *V. rupestris*, allowed the clade consisting of *V. *×* champinii* and *V. mustangensis* to switch position with *V. monticola*, such that BS support for node A dropped to almost zero (Fig. 2e). The close relatives of *V. rupestris* still drew *V. *×* champinii* towards their clade, which prevented *V. mustangensis* from shifting to the base of the ingroup.

(4) Finally, exclusion of the parent taxa rendered the hybrid, *V. *×* champinii*, monophyletic with high BS support (Fig. 2e, Appendix S2).

#### Hybrid populations in *Tropheus moorii* (Cichlidae)

Hybrid genomes were detected in several colour variants of the cichlid fish *Tropheus* by a HET on AFLP data (Egger *et al*. 2007). Two of these cases were pursued by independent population genetic analyses, which confirmed genetic admixture in the putative hybrid populations (Sefc *et al*. 2007; Mattersdorfer 2011). The HET analysis of Egger *et al*. (2007) was replicated with hext as described in Appendix S3.

#### Hybridization among North American wolf‐like Canidae

Details of another example for the use of hext with a large SNP data set are given in Appendix S4. In brief, a HET based on 47 845 SNP genotypes representing 18 taxonomic/geographic groups of wolf‐like canids (vonHoldt *et al*. 2011) gave rise to the expected hybrid signal for the Great Lakes wolf population (Koblmüller *et al*. 2009), with genomic contributions from north‐eastern American grey wolves and coyotes and/or Algonquin Park wolves.

### Simulated Data

#### Hybrid signals

True positives, that is upper BS outliers for clades containing the parental taxa upon exclusion of the hybrid taxon, were observed in analyses of 10 of the 19 data sets that contained a hybrid taxon (Table 2). Additionally, exclusion of a taxon descending from one of the hybrid's parents (‘parent taxon’) caused BS outliers for that taxon's clade in six cases. Hybrid‐caused outliers and parent‐caused outliers mainly identified the same parental lineage, while in one case (simulation #22) exclusions of parent taxa, but not the exclusion of the hybrid taxon, caused BS outlier signals (Table 2).

In two simulations (#19 and #26), the presence of the hybrid taxon interfered with the retrieval of the simulated tree topology (shown in Fig. 3a) and created a novel node in the full tree that joined all ingroup taxa except o, w, x and y. Exclusion of the hybrid taxon caused this node to disappear, which became evident in the boxplots in the form of lower outlier values at BS = 0 for this node.

#### False positives: upper outliers caused by the exclusion of non‐hybrid taxa

**Figure 4:**
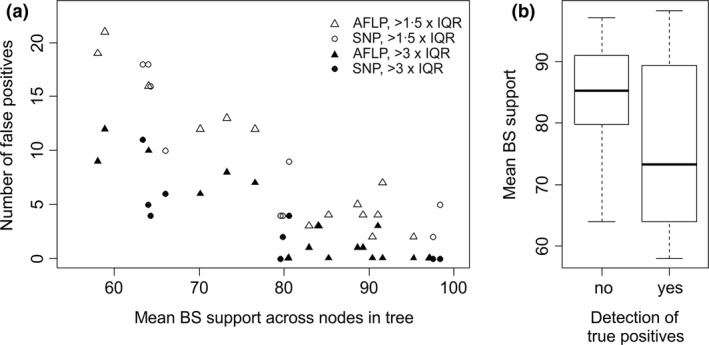
(a) Negative correlation between numbers of false‐positive boxplot outliers (upper BS outliers unconnected to hybrid taxon) and average node support across the full tree in simulations of AFLP and SNP data. Outliers were identified as values at a distance >1·5 × IQR (open symbols) and >3 × IQR (filled symbols) from the third quartile. (b) True positive boxplot outliers (upper BS outliers upon exclusion of hybrid taxon) were more likely to occur when average node support in the full tree was low.

Homoplasy excess tests on the simulated data sets returned a considerable number of false‐positive outlier signals (Table 2). The number of false positives showed a strong negative correlation with average node support across the tree (outliers >1·5 × IQR, *r* = −0·88, *P* = 2·2 × 10^−9^, outliers >3 × IQR, *r* = −0·85, *P* = 4·1 × 10^−8^), with no difference in the relationship between AFLP and SNP data sets (Fig. 4a). Hybrid taxon‐induced outliers in the simulations were not more extreme than false‐positive outliers, such that the stricter outlier criterion (3 × instead of 1·5 × IQR from upper quartile) reduced the true and the false signals alike (false positives from a total of 213 to 93; true positives caused by hybrid exclusion from 17 to 7; Table 2). Similarly, the reduction of the total number of upper outliers in better‐resolved trees (simulated with more loci or smaller population sizes) at the same time increased the probability of missing true hybrids (Table 2).

As in the analysis of empirical data (e.g. *Vitis* example, Fig. 2c), not all of the false positives would be interpreted as indications of hybrid taxa. Most previous applications of the HET (Table 1) did not consider intrataxon nodes in the outlier tests, but focussed on nodes joining different taxa. With appropriate inspection of the hext output and incorporating knowledge about the investigated organisms, for example on their geographical distribution, only a fraction of the upper outliers in the hext output would give rise to false inferences of putative hybrid taxa. For instance, the average number of false positives per simulation dropped from 8 (across all nodes) to 3 when only inner nodes which joined at least two taxa were considered (Table 2), while all true hybrid signals were retained. In the analysis of the *Vitis* data set, this strategy would preclude all signals unrelated to *V. *×* champinii* and its parent species, but also overlook one informative BS outlier for the monophyly of *V. rupestris* (Fig. 2c).

We also examined whether fewer false‐positive upper outliers would occur when the boxplots were based on a larger number of taxon‐exclusion experiments (i.e. more BS values sampled per node). To this aim, we (i) simulated data sets with more taxa (36 ingroup taxa, i.e. 36 taxon‐jackknife trees) and (ii) increased the jackknife sample size to *n* = 48 in data sets with 19 ingroup taxa by additionally excluding pairs of randomly selected individuals (Appendix S5); the latter approach has been taken in several empirical applications of the HET (Table 1). In both cases, the numbers of false positives rose considerably in comparison with analyses with fewer taxon‐jackknife trees (Appendix S5).

False‐positive outliers originate from heterogeneity in shared ancestral polymorphism across taxon pairs that is not due to hybridization. Levels of divergence differed among simulated taxa, and we observed that the exclusion of a taxon which belonged to a weakly resolved clade of closely related taxa frequently led to upper outlier BS values for nodes joining other taxa in this clade. However, even when all taxa were approximately equally divergent, the HET returned upper outliers at several inner nodes (simulation #7 in Table 2), probably as a consequence of lineage sorting stochasticity.

#### False negatives: no upper outliers after exclusion of hybrid taxon

Exclusion of the hybrid taxon failed to produce an expected boxplot outlier signal in nine of the 19 simulations that included a hybrid taxon. In particular, upper outlier signals almost never identified both parental lineages (Table 2). False negatives were more frequent in strongly than in weakly supported trees (Fig. 4b), but the difference was not significant (*F*_1,17_ = 2·22, *P* = 0·15).

False negatives in the HET can occur for a number of reasons. There are situations in which it is *a priori* impossible for the HET to detect a hybrid signal. If bootstrap support in the tree is high despite the presence of a hybrid taxon, there is simply no room for HET outlier signals. High bootstrap support in the full tree prevented the detection of one parent especially in simulations of more ancient hybridization (#16, #24, #25, #26; Table 2), or when the number of loci in simulations of a given hybridization scenario was increased (Table 2). Furthermore, accounting for support carryover (see ‘Overview of hext’) precludes a HET outlier signal if the hybrid is placed as sister to its parental lineage in the full tree (e.g. #14).

HET boxplot outliers can also fail to appear when the presence of the hybrid taxon changes the topology of the tree. Exclusion of the hybrid taxon may then cause a dramatic rise of BS support for the ‘true’ node, but as HET boxplot graphs only include nodes that are present in the full tree, this type of hybrid signal would go unnoticed unless it is accompanied by a simultaneous severe drop in BS support for one of the original nodes (as in the *Vitis* example and in simulations #19 and #26 in Table 2). The program hext outputs all taxon‐jackknife trees and stores information on all nodes occurring in the full and jackknife trees, allowing the user to scrutinize the topologies of the taxon‐jackknife trees and to retrieve BS values for alternative nodes (not present in full tree) across taxon‐jackknife analyses.

False negatives also occurred with simulated data sets underlying weakly supported trees, when high background levels of shared polymorphism among taxa kept BS support low even when the hybrid taxon was excluded from the tree (simulations #8, #13).

Finally, exclusion of the hybrid taxon often caused BS values for clades including a parent to reach their maximum value, but without becoming an outlier in the boxplot (Fig. 3b; Table 2). Boxplots tend to overlook outlier values in small samples (Seo 2006). However, increasing the sample size of BS values per node by randomly excluding pairs of individuals in addition to the taxonwise exclusions did not improve the detection rate of hybrids (Appendix S5).

## Conclusions

The present study ascertains the functionality of a new software program automating a taxon‐jackknife‐based approach to hybrid detection. Analyses of empirical data corroborated putative hybrid taxa. Importantly, the origin of *V. *× *champinii* from hybridization has not been tested with genetic data prior to this study and was supported by several consistent signatures in the taxon‐jackknife trees.

In the simulations, upper boxplot outliers did not reliably indicate hybrid taxa. In particular, the high numbers of false positives among the upper outliers in some of the simulations suggest that applications of the HET to empirical data might sometimes overestimate the occurrence of hybrid taxa if inferences were based on boxplot outliers alone. Conversely, false negatives in the analyses of simulated data sets imply that a lack of HET signals does not prove the absence of hybrid taxa in the phylogeny. False negatives were particularly frequent in the simulations of the most ancient hybrid taxa (#22–26 in Table 2). For the more recent hybrid taxa in simulations #8–21, the best results in terms of true vs. false positives were obtained when average BS support for the tree was >70%, the outlier criterion was set to >1·5 × IQR, and only nodes joining at least two species were considered.

Previous studies using the HET have often supplemented the information derived from boxplot outliers with analyses of cyto‐nuclear discordance (Schliewen & Klee 2004; Schwarzer, Misof & Schliewen 2012a; Schwarzer *et al*. 2012b; Weiss, Cotterill & Schliewen 2015), split decomposition (Kidd, Kidd & Kocher 2006), and branch attachment frequencies (Schwarzer, Misof & Schliewen 2012a; Schwarzer *et al*. 2012a), as well as considered the distribution and biology of the investigated taxa (Herder *et al*. 2006; Egger *et al*. 2007; Larsen, Marchán‐Rivadeneira & Baker 2010), and have followed up on the inferred hybrid taxa with population genetic analyses (Larsen, Marchán‐Rivadeneira & Baker 2010; Mattersdorfer 2011). Combining evidence from different approaches to hybrid detection is strongly recommended to reduce the risk of spuriously claiming hybrid taxa.

Beyond the test for bootstrap outliers, taxon‐jackknifing produces valuable information, which can be scrutinized for signatures of hybridization (e.g. Lucek *et al*. 2010). These signatures include changes in tree topology across taxon‐jackknife trees, consistent effects of exclusions of the putative hybrid taxon and of its putative parent taxa, and maximum (but not outlier) BS values upon the exclusion of a suspected hybrid taxon. Furthermore, nodes that joined the hybrid taxon or the parental lineages to the tree often had particularly wide variances in bootstrap support across jackknife trees, evident as boxplots with wide boxes and whiskers but not necessarily with outliers (Fig. 3b). To the researcher, this pattern points out nodes that were affected by the exclusion of several taxa and might merit further attention.

The detection of these rather complex effects of hybrid taxa in taxon‐jackknife experiments requires careful scrutiny of the taxon‐jackknife trees and appears inaccessible to full automation. The software hext supports the taxon‐jackknife approach to hybrid detection by automating the jackknifing procedure, parsing the output of the bootstrap analyses, and providing conveniently assembled summaries as well as access to all tree data collected during the analysis.

## Funding

The AFLP data were generated with support from the Austrian Science Fund (FWF), grant numbers P20883 and P17380 to KMS.

## Data accessibility

The Tropheus AFLP data set and the canid SNP data (vonHoldt *et al*. 2011) used here are available at DRYAD entry doi:10.5061/dryad.20t14. The Vitis SNP data of Miller *et al*. 2013 were retrieved from dryad (http://dx.doi.org/10.5061/dryad.4181f), and the Vitis accessions used in our analysis are listed in Appendix S6. The hext program, user manual and example data files are available on http://www.uni-graz.at/~sefck/HExT.zip.

## Supporting information


**Appendix S1.** A list and description of functions in the hext program.

**Appendix S2.** Hybrid signal in a SNP‐based phylogeny of the genus *Vitis* (grape and relatives).

**Appendix S3.** Hybrid populations in *Tropheus moorii* (Cichlidae).

**Appendix S4.** Hybridization among North American wolf‐like Canidae.

**Appendix S5.** HET results with simulated datasets.
