# Supplementary material for: hext, a software supporting tree‐based screens for hybrid taxa in multilocus data sets, and an evaluation of the homoplasy excess test
Source: Methods Ecol Evol. 2015 Nov 11;7(3):358–68. doi: 10.1111/2041-210X.12490 (PMC4824276; doi:10.1111/2041-210X.12490)
Supplement: Supplementary file 1 — Appendix S1. A list and description of functions in the hext program. [file MEE3-7-358-s001.docx]

**Appendix S1 to ‘HExT, a software supporting tree-based screens for hybrid taxa in multi-locus datasets, and an evaluation of the homoplasy excess test’ by K. Schneider et al.**

**A list and description of functions in the HExT program.**

| ***function*** | ***description*** |
| --- | --- |
| access_alttop.r | accesses BS support information from alternative topologies |
| access_custom | enables customized BS support queries; displays and (if desired) stores query results |
| all_descendants | returns the descending taxa for each clade of an input tree |
| alttop_search | searches for topologies of bootstrap trees not present in the full tree, sorts the obtained data frame, and stores it in a file |
| boot_excl | determines sister nodes and those that are lost in the course of jackknifing |
| descendants | determines the descending tips of a specified node |
| excl_spec | writes jackknife sets to a file |
| fas_parse | exctracts data and sample names from an input fasta file |
| file_input | calls input parsing functions on the basis of input file extensions |
| geno_parse | extracts the SNP genotype data and sample names from an input text file |
| get_trees | performs jackknifing and calls functions for tree construction and for obtaining tree bipartition information |
| inp_manip | splits specified jackknife sets or outgroups into taxon names |
| input_mod | parses input from the “interact” function |
| interact | accepts input and parameter values from the user |
| make_bin | converts an integer genotype matrix to four binary matrices |
| nex_parse | extracts data and sample names from an input nexus file |
| njf | calls tree construction and rooting functions |
| node_comp | compares tree topologies and collects BS support data |
| outlier_sink | stores boxplot outlier information in a text file and additional boxplots |
| parse_trees | organizes comparison of full and jackknifed trees, obtains BS support for trees and organizes storage of tree topologies |
| plot_manip | manipulates boxplots from the “access_custom” function |
| rowcol_names | assigns row and column names to the input matrix |
| seq_dist | calculation of Nei-Li or allele sharing distance matrices |
| sisternodes | determines sister nodes of jackknifed clades |
| store_plot | creates BS boxplots and stores these and the underlying BS information in pdf and txt files, respectively |
| tax_comp | collects taxa for jackknifing in a vector |
| write_nodes | writes descending taxa and BS support values of all nodes of all trees to a txt file |
| write_par | writes parameter information of HExT runs to a txt file |
