# Supplementary material for: hext, a software supporting tree‐based screens for hybrid taxa in multilocus data sets, and an evaluation of the homoplasy excess test
Source: Methods Ecol Evol. 2015 Nov 11;7(3):358–68. doi: 10.1111/2041-210X.12490 (PMC4824276; doi:10.1111/2041-210X.12490)
Supplement: Supplementary file 2 — Appendix S2. Hybrid signal in a SNP‐based phylogeny of the genus Vitis (grape and relatives). [file MEE3-7-358-s002.docx]

**Appendix S2 to ‘HExT, a software supporting tree-based screens for hybrid taxa in multi-locus datasets, and an evaluation of the homoplasy excess test’ by K. Schneider et al.**

**Hybrid signal in a SNP-based phylogeny of the genus *Vitis* (grape and relatives)**

The following figures S2.1 – S2.4 show NJ trees based on the full data and on selected taxon-jackknife data.





Figure S2.1. The full tree.





Fig. S2.2. Taxon-jackknife tree: *V. x champinii* excluded from dataset.





Fig. S2.3. Taxon-jackknife tree: *V. mustangensis* excluded from dataset.





Fig. S2.4. Taxon-jackknife tree: *V. rupestris* excluded from dataset.
