# Supplementary material for: hext, a software supporting tree‐based screens for hybrid taxa in multilocus data sets, and an evaluation of the homoplasy excess test
Source: Methods Ecol Evol. 2015 Nov 11;7(3):358–68. doi: 10.1111/2041-210X.12490 (PMC4824276; doi:10.1111/2041-210X.12490)
Supplement: Supplementary file 3 — Appendix S3. Hybrid populations in Tropheus moorii (Cichlidae). [file MEE3-7-358-s003.docx]

**Appendix S3 to ‘HExT, a software supporting tree-based screens for hybrid taxa in multi-locus datasets, and an evaluation of the homoplasy excess test’ by K. Schneider et al.**

**Hybrid populations in *Tropheus moorii* (Cichlidae)**

Hybrid genomes were detected in several color variants of the cichlid fish *Tropheus* by a HET on AFLP data (Egger et al. 2007). Two of these cases were pursued by independent population genetic analyses, which confirmed that the putative hybrid populations represented genetic admixtures of the putative parent lineages identified by HET (Sefc et al. 2007; Mattersdorfer 2011).

The HET analysis of Egger et al. (2007) was replicated with HExT as follows.

The dataset used in the HET comprised 117 samples representing 51 populations of the cichlid fish genus *Tropheus* from Lake Tanganyika. 56 taxon-jackknife trees were calculated by excluding one population at a time and, additionally, by excluding groups of populations.

The full tree had 116 nodes. Following Egger et al. (2007) by setting the boxplot outlier criterion to >3 times IQR, a total of 93 upper outliers were detected at a total 54 nodes (>1 upper outlier at some nodes; Fig. S3.1). Egger et al. (2007) focussed on 21 nodes that defined the various *Tropheus* color variants as well as putative and described species, and examined BS values for these nodes across taxon-jackknife trees. HExT output revealed a total of 34 upper outliers (>3 x IQR) at 15 of these nodes (Fig. S3.2). In comparison to the HET by Egger et al. (2007), BS values for nodes that might benefit from support carryover after exclusion of their sister taxon are not included in the outlier test done by HExT.

Egger et al. (2007) then examined each of the detected outliers. mtDNA data, color pattern phenotypes as well as geographic distribution of suggested hybrid and parent taxa were considered in the interpretation of the HET signal. Five putative hybrid taxa (populations and color variants) were inferred. The annotated boxplots supporting this inference (a subset of the boxplots in Fig. S3.2) are shown in Fig. S3.3.

The HET-indicated hybrid taxa whose mosaic genomes were confirmed by independent population genetic approaches are the ‘yellow morph near Lufubu estuary’ and ‘Katoto’ (Sefc et al. 2007; Mattersdorfer 2011). The interpretation of the boxplot diagrams is explained on the basis of the results obtained by exclusion of the yellow morph (second panel from top in Fig. S3.3). Four populations in the dataset represented the yellow morph: Lufubu, Ilangi, Inangu and Kabeyeye. Jackknife sets comprised samples from each of these populations at a time, a fifth jackknife set combined samples from all four populations, and a sixth jackknife set combined the four populations with samples from Livua. Marked increases in BS support were observed at nodes 1, 1a, 2, and 4a (node labels as in Egger et al. 2007) in one or more of the resulting jackknife trees. Node 2, however, defines a sister clade of the excluded samples, and is therefore ignored by HExT (for comparison with Egger et al., the outlier value at this node is marked by ‘SC’ in Fig. S3.3). Nodes 1 and 1a (nested in node 2) join bluish-coloured populations occurring south of the yellow morph, and node 4b joins red-coloured populations occurring north. This suggests the yellow morph as hybrid taxon, with the bluish and the red *Tropheus* as the two parents.

For interpretations of the remaining examples in Fig. S3.3, we refer to the original publication of Egger et al. (2007; see also Additional file 2 in that publication).

**References:**

Egger, B., Koblmüller, S., Sturmbauer, C. & Sefc, K.M. (2007) Nuclear and mitochondrial data reveal different evolutionary processes in the Lake Tanganyika cichlid genus *Tropheus*. *BMC Evolutionary Biology*, 7, 137.

Mattersdorfer, K. (2011) The evolution of phenotypic diversity in a Lake Tanganyika cichlid fish. Dissertation thesis, University of Graz, Austria.

Sefc, K.M., Baric, S., Salzburger, W. & Sturmbauer, C. (2007) Species-specific population structure in rock-specialized sympatric cichlid species in Lake Tanganyika, East Africa. *Journal of Molecular Evolution*, 64, 33-49.





**Figure S3.1. Boxplots of BS values for all nodes at which upper outlier values >3 x IQR beyond the third quartile were observed (‘upper outlier boxplots’)**. The plot is standard output of HExT (file ‘upper outlier boxplots’), except that node labels used in Egger et al. (2007) were added to the node numbers on the y-axis. Open circles represent outlier values. Not all of the nodes investigated in Egger et al. are shown in this plot, because not all of them had upper outliers.


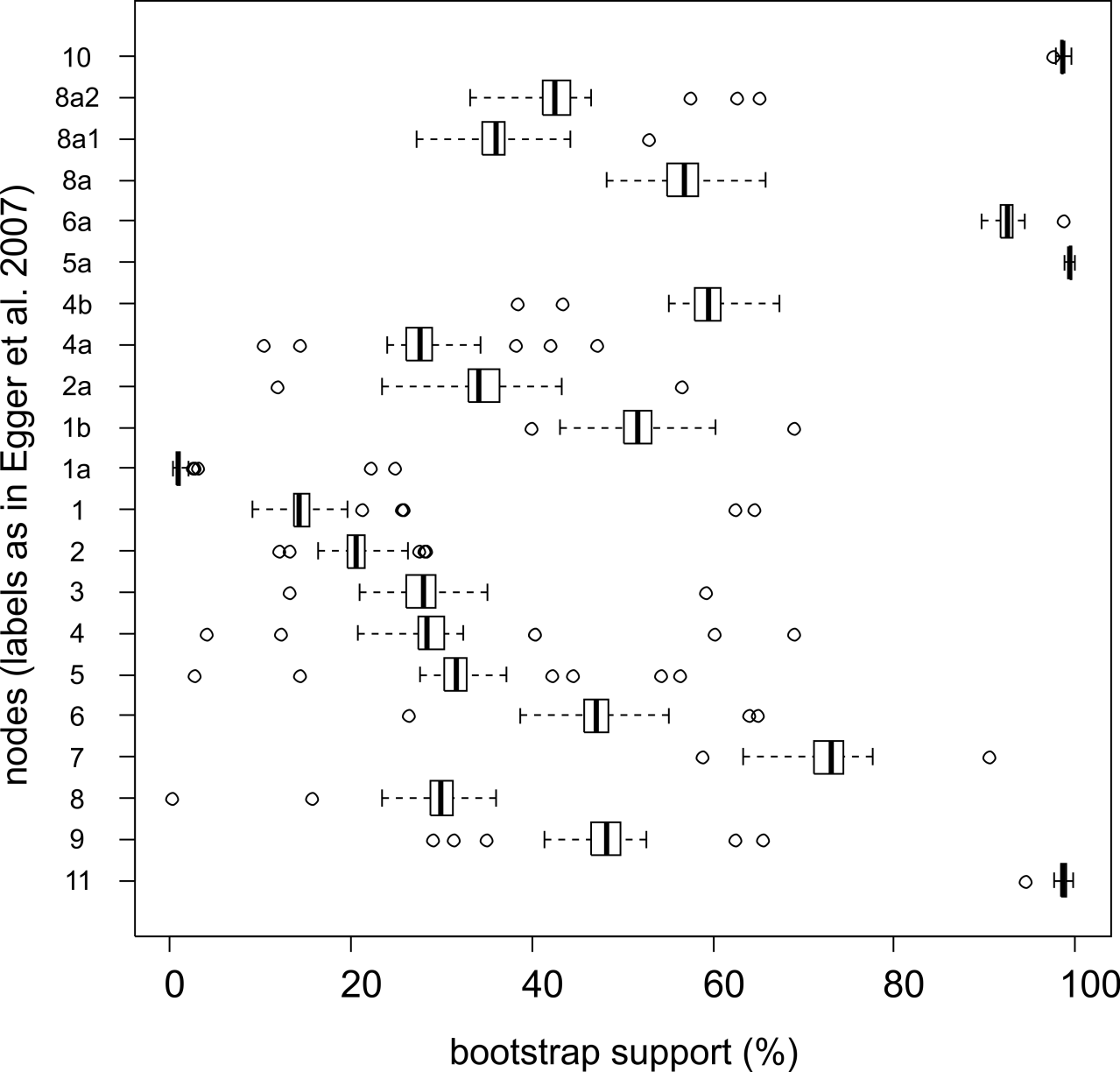


**Figure S3.2. Boxplots of BS values for the nodes investigated in Egger et al. (2007).** Nodes are labelled as in Egger et al. Open circles represent outlier values >3 x IQR beyond the third quartile. Most, but not all of the investigated nodes had upper outliers, and many nodes had more than one upper outlier.

This plot can be drawn within HExT as part of the CUSTOM analysis. HExT allows the user to request boxplots for selected nodes, which are defined by the node labels used in the output tree ‘trees_numlabels.nwk’.

Alternatively, custom boxplots can be drawn based on the HExT output table ‘bootstrap_table.txt’ in any statistics program. Here, we used the R command

boxplot(x=data[-1,c(node1, node2, node3, etc...)], range=3, horizontal=TRUE, las=1, cex.axis=0.5, ylab = "nodes (labels as in Egger et al. 2007)", xlab="bootstrap support (%)")

with node1, node2, etc… replaced by the node labels of the requested nodes,

and changed the node labels to correspond to Egger et al. in CorelDraw.





**Figure S3.3. Boxplots, which were interpreted to indicate hybrid taxa by Egger et al. (2007)** (a subset of the boxplots of Figure S3.2). Each of the five panels addresses one inferred hybrid taxon, whose exclusion gave rise to upper outlier BS values at one or more nodes. Outlier BS that occurred in trees excluding the indicated taxon are shown as filled circles. More than one filled circle at a node can occur when the excluded taxon is represented in more than one taxon-jackknife set. For example, in panel two, upper outliers at node 1 are caused by exclusion of all four yellow-morph populations, by excluding them together with population Livua, and by separately excluding Ilangi and Inangu, respectively. The latter two outlier dots overlap in the plot at BS=25.7% and BS=25.6%.

Outlier values occurring upon exclusions of taxon-jackknife sets that do not include the investigated taxa are shown as open circles. These could be false positives (no hybridization underlying the outlier signal) or true signals (i.e. caused by mosaic genomes due to hybridization) that could not be interpreted conclusively.

SC marks outlier BS values at nodes that might have benefitted from support carryover after exclusion of a sister taxon. These values are included in the plot only for comparison with the boxplots in Figure 4 in Egger et al. (2007), which had not omitted these cases.

BS support for the nodes in the full tree is shown on the right side of the boxplot panels.
