# Supplementary material for: hext, a software supporting tree‐based screens for hybrid taxa in multilocus data sets, and an evaluation of the homoplasy excess test
Source: Methods Ecol Evol. 2015 Nov 11;7(3):358–68. doi: 10.1111/2041-210X.12490 (PMC4824276; doi:10.1111/2041-210X.12490)
Supplement: Supplementary file 4 — Appendix S4. Hybridization among North American wolf‐like Canidae. [file MEE3-7-358-s004.docx]

**Appendix** **S4 to ‘HExT, a software supporting tree-based screens for hybrid taxa in multi-locus datasets, and an evaluation of the homoplasy excess test’ by K. Schneider et al.**

**Hybridization among North American wolf-like Canidae**

Hybridization played an important role in the evolution of wolf-like canids, especially in North America (e.g. Koblmüller *et al*. 2009; Kays *et al*. 2010; Bohling & Waits 2011; vonHoldt *et al*. 2011). For example, wolves of the Great Lakes region (hereafter GL wolves) are predominantly of grey wolf (*Canis lupus*) ancestry, with introgression from coyote (*Canis latrans*) and/or from the wolf population from Algonquin Park (Koblmüller *et al*. 2009; Fain *et al*. 2010; vonHoldt *et al*. 2011). Since the taxonomic classification of the wolves from Algonquin Park as either a subspecies of the grey wolf (*C. lupus lycaon*) or a separate species (eastern wolf *C. lycaon*) is unresolved, we refer to this population as the ‘Algonquin Park wolf’.

Using the large SNP dataset generated by vonHoldt *et al*. (2011), we tested the ability of HExT to retrieve a hybrid signal for the Great Lakes wolf population. 47,845 SNP genotypes representing 18 taxonomic/geographic groups of wolf-like canids were derived from the Affymetrix Canine SNP Genome Mapping Array (48kSNP; vonHoldt *et al*. 2011). In HExT, 1000 bootstrap replicates were calculated with the full and with each taxon-jackknife dataset. For taxon-jackknifing, one taxonomic/geographic group at a time was removed from the dataset (jackknife sets are listed in Table S4.1 below). The golden jackal (*C. aureus*) was used as outgroup.

In the full tree, most taxonomic/geographic groups were monophyletic with 100% BS support (Fig. S4.1A, Fig. S4.2), and phylogenetic relationships among the taxonomic/geographic groups were usually well supported, leaving little room for increases of BS support in response to the exclusion of a hybrid taxon. With regard to GL wolf ancestry, 100 % BS support for coyote and for the Algonquin Park wolf in the full tree a priori precluded a response to hybrid exclusion at these nodes. Nonetheless, several changes in BS support and tree topology upon taxon-jackknifing supported the presumed mixed ancestry of GL wolves:

(1) In the full tree, GL wolves were placed within a weakly supported clade of northern American grey wolves (node B in Fig. S4.1A). Exclusion of the GL wolves increased BS support for this clade from 68% to 100%, producing an outlier in the BS boxplot (Fig. S4.1D). This is consistent with the presumed origin of the GL wolves, as homoplasy due to introgression from coyote and eastern wolf is expected to reduce BS support for a placement of GL wolves within the grey wolf clade.

(2) Exclusions of groups within the northern American grey wolf clade informed on the grey wolf ancestry of GL wolves. When north-eastern American grey wolves were excluded from the dataset, GL wolves appeared basal to all remaining grey wolves (Fig. S4.1B) and BS support for nodes joining GL wolves with American grey wolves dropped to 1% (nodes A, B and C in Fig. S4.1A). In contrast, exclusion of north-western American grey wolves did not affect the position of GL wolves in the tree, but increased BS support for a sistergroup relationship between GL wolves and north-eastern American grey wolves from 82% to 100% (Fig. S4.1C). This suggests that GL wolves were drawn into the northern American grey wolf clade by the north-eastern American grey wolves, whereas exclusion of this group allowed GL wolves to assume a position closer to their other hybrid parents (coyote and Algonquin Park wolf).

The dataset contains additional putatively admixed taxa such as north-eastern coyotes, red wolves (*C. rufus*), and the Algonquin Park wolves. However, in these cases, the presumed parental taxa (vonHoldt *et al*. 2011) were either placed as sistergroup of the hybrid taxa or within well-supported clades, both of which precluded the identification of a hybrid signal in the HET.

***References***

Bohling JH, Waits LP (2011) Assessing the prevalence of hybridization between sympatric *Canis* species surrounding the red wolf (*Canis rufus*) recovery area in North Carolina. *Molecular Ecology*, **20**, 2142-2156.

Fain SR, Straughan DJ, Taylor BF (2010) Genetic outcomes of wolf recovery in the western Great Lakes. *Conservation Genetics*, **11**, 1747-1765.

Kays R, Curtis JJ, Kirchman JJ (2010) Rapid adaptive evolution of northeastern coyotes via hybridization with wolves. *Biology Letters*, **6**, 89-93.

Koblmüller S, Nord M, Wayne RK, Leonard JA (2009) Origin and status of the Great Lakes wolf. *Molecular Ecology*, **18**, 3508-3517.

vonHoldt BM, Pollinger JP, Earl DA **et al.** (2011) A genome-wide perspective on the evolutionary history of enigmatic wolf-like canids. *Genome Research*, **21**, 1294-1305.

**Figure S4.1.** HET hybrid signal for the Great Lakes wolf. Numbers at tree nodes are BS values. (A) The full tree. (B) Tree obtained after exclusion of the grey wolf populations from north-eastern America. (C) Tree obtained after exclusion of the grey wolf populations from north-western America. (D) Bootstrap outlier at the North American grey wolf clade (node B) produced by the exclusion of the Great Lakes wolves.


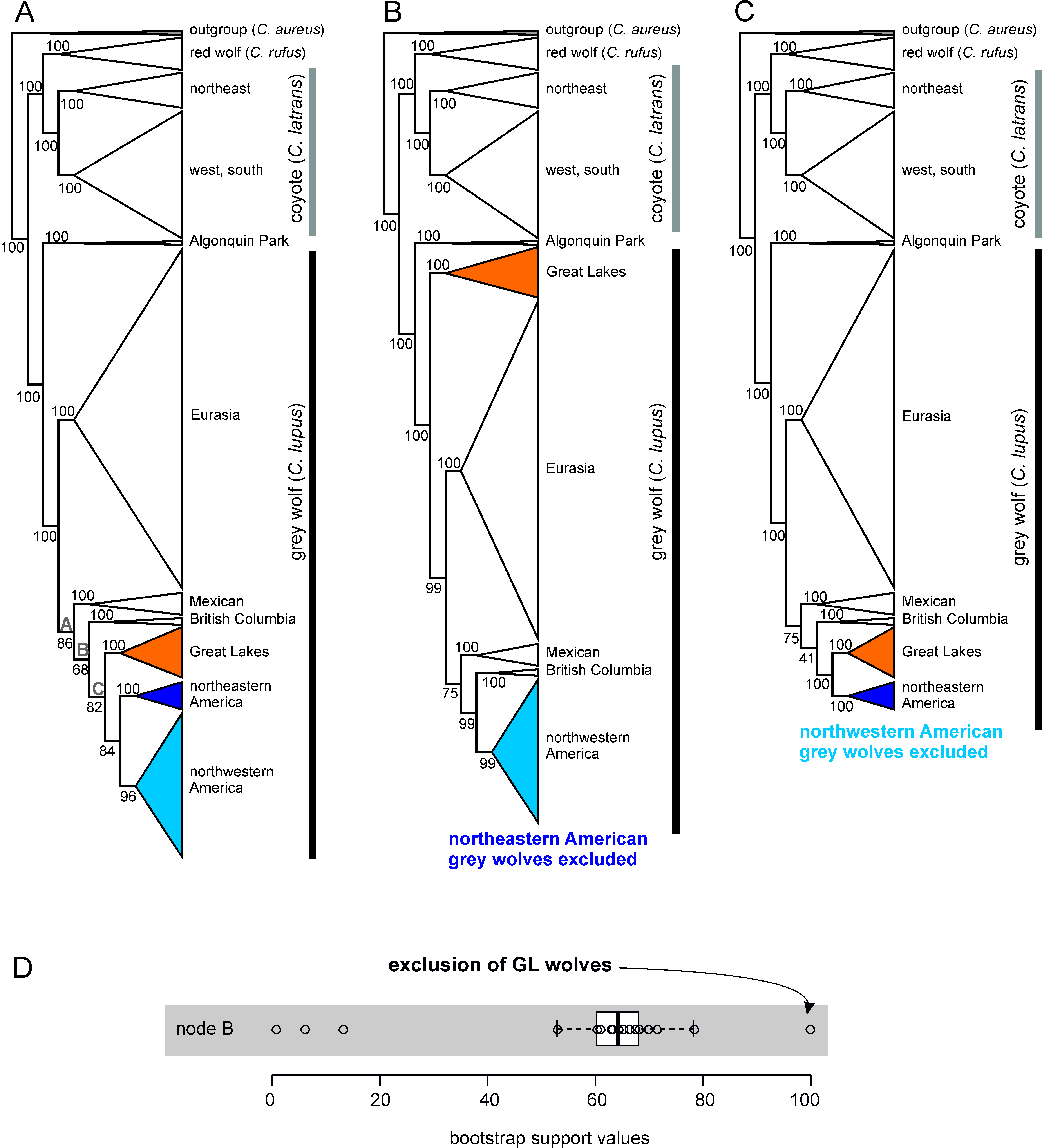


**Figure S4.2** Neighbor Joining tree of the Canidae dataset.





**Table S4.1.** Taxon jacknife sets for the canid HET. Sample names refer to the sample IDs in the SNP dataset of vonHoldt et al. (2011).

| **Jackknife set** | **Taxon specification** | **IDs of excluded samples** |
| --- | --- | --- |
| 1 | Northeastern coyotes | 11396_CLA_NH 11397_CLA_VT 11398_CLA_NY 11400_CLA_NY 11401_CLA_NY 11403_CLA_VT 11404_CLA_NY 11405_CLA_NY 11406_CLA_CT 2709_CLA_NY 2715_CLA_QUE 2717_CLA_NY 2718_CLA_NY |
| 2 | Chinese wolves | 3535_CLU_CHINA 3536_CLU_CHINA W01_CLU_CHINA W04_CLU_CHINA W05_CLU_CHINA W06_CLU_CHINA W07_CLU_CHINA W08_CLU_CHINA W09_CLU_CHINA W10_CLU_CHINA |
| 3 | Northeastern European wolves | 10115_CLU_BELA 11302_CLU_BELA 10103_CLU_BELA 10126_CLU_BELA 10117_CLU_BELA 10119_CLU_BELA 10125_CLU_BELA 10142_CLU_LITH 11327_CLU_POLN 10134_CLU_POLN 10129_CLU_POLN 10135_CLU_POLN 11335_CLU_POLN 11359_CLU_POLN 11329_CLU_POLN 10099_CLU_POLN 11305_CLU_RUSS 11316_CLU_RUSS 11317_CLU_RUSS 11330_CLU_RUSS 11331_CLU_RUSS 11332_CLU_RUSS 11333_CLU_RUSS 11342_CLU_RUSS 11343_CLU_RUSS 11318_CLU_RUSS 10128_CLU_RUSS 10123_CLU_RUSS 10132_CLU_RUSS 10139_CLU_RUSS 10130_CLU_RUSS 10146_CLU_RUSS 10121_CLU_RUSS 10124_CLU_RUSS 11306_CLU_SLOV 11307_CLU_SLOV 11334_CLU_SLOV 2972_CLU_SWEDEN 2998_CLU_SWEDEN 11348_CLU_UKR 11315_CLU_UKR 11346_CLU_UKR 11347_CLU_UKR 10127_CLU_UKR 10131_CLU_UKR 10108_CLU_UKR 10137_CLU_UKR 10122_CLU_UKR 11313_CLU_UKR |
| 4 | Balkan wolves | 11310_CLU_BULG 11320_CLU_BULG 11328_CLU_BULG 3919_CLU_CROAT 3920_CLU_CROAT 3941_CLU_GREECE |
| 5 | Italian wolves | 2730_CLU_ITALY 2753_CLU_ITALY 2755_CLU_ITALY 2756_CLU_ITALY 2794_CLU_ITALY 2725_CLU_ITALY 2781_CLU_ITALY 2795_CLU_ITALY 2797_CLU_ITALY 2799_CLU_ITALY 2800_CLU_ITALY 11355_CLU_ITALY 11353_CLU_ITALY 11354_CLU_ITALY 2747_CLU_ITALY 2785_CLU_ITALY 2729_CLU_ITALY 2742_CLU_ITALY 2748_CLU_ITALY 2751_CLU_ITALY |
| 6 | Iberian wolves | 11246_CLU_SPAIN 11248_CLU_SPAIN 11252_CLU_SPAIN 11253_CLU_SPAIN 11255_CLU_SPAIN 11249_CLU_SPAIN 11250_CLU_SPAIN 11251_CLU_SPAIN 11256_CLU_SPAIN |
| 7 | Mexican wolves | 403_CLU_MEX_Gr 404_CLU_MEX_Gr 2540_CLU_MEX_Ar 11217_CLU_MEX_SB 11214_CLU_MEX_SB 2535_CLU_MEX_Ar 405_CLU_MEX_Gr 11215_CLU_MEX_SB 11216_CLU_MEX_SB |
| 8 | Algonquin Park wolves | 11295_CLU_ALGON 11297_CLU_ALGON |
| 9 | Red wolves | 11142_CRU 9721_CRU 11143_CRU 9722_CRU 9725_CRU 11140_CRU 9701_CRU 11137_CRU 9700_CRU 9708_CRU 9723_CRU 11141_CRU |
| 10 | Northeastern American wolves | 9496_CLU_NQUE-I 9499_CLU_NQUE-I 11232_CLU_NQUE-I 11233_CLU_NQUE-I 11234_CLU_NQUE-I 9510_CLU_NQUE-U 9511_CLU_NQUE-U 9512_CLU_NQUE-U 9515_CLU_NQUE-U 9502_CLU_NQUE-U 11228_CLU_ONT |
| 11 | Northwestern American wolves | 9558_CLU_AK 9560_CLU_AK 9561_CLU_AK 9557_CLU_AK 9550_CLU_CANADA 6508_CLU_CANADA-FB 6484_CLU_CANADA-FB 6501_CLU_CANADA-FB 2042_CLU_CANADA-FBG 6509_CLU_CANADA-FBG 2043_CLU_CANADA-FG 2056_CLU_CANADA-FG 3132_CLU_CANADA-FG 6505_CLU_CANADA-FG 2048_CLU_CANADA-FW 2049_CLU_CANADA-FW 2052_CLU_CANADA-FW 6515_CLU_CANADA-FW 6499_CLU_CANADA-TaB 2352_CLU_CANADA-TaB 2223_CLU_CANADA-TaW 2297_CLU_CANADA-TaW 2363_CLU_CANADA-TaW 2131_CLU_CANADA-TuG 2130_CLU_CANADA-TuG 2148_CLU_CANADA-TuG 2135_CLU_CANADA-TuGR 2142_CLU_CANADA-TuW 2156_CLU_CANADA-TuW 2144_CLU_CANADA-TuW 2155_CLU_CANADA-TuW 9732_CLU_YNP 9731_CLU_YNP 9734_CLU_YNP 9733_CLU_YNP 10079_CLU_YNP 10080_CLU_YNP 10081_CLU_YNP 10082_CLU_YNP 10083_CLU_YNP 10085_CLU_YNP 10088_CLU_YNP 10092_CLU_YNP 10095_CLU_YNP 10096_CLU_YNP 10097_CLU_YNP 10098_CLU_YNP 9736_CLU_YNP 9735_CLU_YNP 9610_CLU_MN |
| 12 | Great Lakes wolves | 145_CLU_IRNP 147_CLU_IRNP 148_CLU_IRNP 2455_CLU_MN 2518_CLU_MN 2458_CLU_MN 2462_CLU_MN 2515_CLU_MN 2523_CLU_MN 2524_CLU_MN 9606_CLU_MN 11218_CLU_MN 11219_CLU_MN 11226_CLU_ONT 2935_CLU_WI 2937_CLU_WI 2947_CLU_WI 2955_CLU_WI |
| 13 | Western & southern coyotes | 2430_CLA_IL 2431_CLA_IL 3025_CLA_UT 3112_CLA_AK 3456_CLA_AK 2427_CLA_IL 2437_CLA_IL 2555_CLA_VA 2556_CLA_VA 3079_CLA_MANIT 3080_CLA_MANIT 3090_CLA_AL 3091_CLA_AL 3094_CLA_MANIT 7092_CLA_CA 7094_CLA_CA 7095_CLA_CA 11274_CLA_WA 7096_CLA_CA 7105_CLA_CA 7107_CLA_CA 9472_CLA 9478_CLA 9486_CLA 9669_CLA 3087_CLA_LA 3088_CLA_LA 2422_CLA_IL 2549_CLA_VA 2554_CLA_VA 11394_CLA_OH 11399_CLA_OH 11395_CLA_OH 3043_CLA_UT 3081_CLA_MANIT 3085_CLA_MS? 3086_CLA_MS? 3089_CLA_LA 3105_CLA_MANIT 11270_CLA_WA 11267_CLA_WA 11271_CLA_WA 7097_CLA_CA 7109_CLA_CA |
| 14 | Arabian wolves | 3943_CLU_ARABIA 3944_CLU_ARABIA 3945_CLU_ARABIA 3947_CLU_ARABIA 3950_CLU_ARABIA 3076_CLU_OMAN 3077_CLU_OMAN 3078_CLU_OMAN |
| 15 | Israelian wolves | 9938_CLU_ISRAEL 9949_CLU_ISRAEL 9953_CLU_ISRAEL 9945_CLU_ISRAEL 9940_CLU_ISRAEL 9959_CLU_ISRAEL 9937_CLU_ISRAEL 9941_CLU_ISRAEL |
| 16 | Indian wolves | 5398_CLU_INDIA 5400_CLU_INDIA 9659_CLU_INDIA |
| 17 | Iranian wolves | 3073_CLU_IRAN 3066_CLU_IRAN |
| 18 | Turkish wolf | 10141_CLU_TURKEY |
| 19 | British Columbia wolves | 3757_CLU_BC 3755_CLU_BC 3756_CLU_BC |
