# Supplementary material for: hext, a software supporting tree‐based screens for hybrid taxa in multilocus data sets, and an evaluation of the homoplasy excess test
Source: Methods Ecol Evol. 2015 Nov 11;7(3):358–68. doi: 10.1111/2041-210X.12490 (PMC4824276; doi:10.1111/2041-210X.12490)
Supplement: Supplementary file 5 — Appendix S5. HET results with simulated datasets. [file MEE3-7-358-s005.docx]

**Appendix S5 to ‘HExT, a software supporting tree-based screens for hybrid taxa in multi-locus datasets, and an evaluation of the homoplasy excess test’ by K. Schneider et al.**

Table S5.1. HETs with simulated datasets.

HETs on datasets containing 36 taxa (no hybrid taxa) were conducted by excluding each taxon at a time. HETs on datasets containing 19 taxa (including one hybrid taxon) are re-analyses of data in Table 2 of the main text and were conducted by excluding each taxon at a time and, additionally, each of 29 jackknife sets that were composed of two random individuals representing two different taxa. The random jackknife sets are given below^1^.

Columns headed ‘simulated datasets’ provide information on simulations and resulting trees (θ, MCcoal population size parameter; hybrid τ, MCcoal divergence time parameter for the origin of the hybrid taxon; mean BS, average bootstrap values for nodes in the full tree). Columns headed ‘upper outliers upon exclusion of hybrid taxon’ report the number of nodes at which upper BS were observed upon exclusion of the hybrid taxon (IQR, inter-quartile range). Columns headed ‘false positive upper boxplot outliers’ report the total number of BS outliers upon exclusion of non-hybrid taxa. The last column refers to simulations in Table 2 of the main text, that are comparable to the one reported in this table (for datasets with 36 taxa) or use the same simulated data (datasets with 19 data).

| Simulated datasets | | | | Upper outliers upon exclusion of hybrid taxon | | False positive upper outliers | | Compare to Table 2 |
| --- | --- | --- | --- | --- | --- | --- | --- | --- |
| Marker loci | θ | Hybrid τ | Mean BS | >1.5 x IQR (indicated parent lineage) | >3 x IQR | >1.5 x IQR | >3 x IQR |  |
| **36 INGROUP TAXA; 36 TAXON-JACKKNIFES; NO HYBRID TAXA** | | | | | | | | |
| 1022 SNP | 0.0005 | n.a. | 57.64 | n.a. | n.a. | 57 | 30 | #3 |
| 8661 AFLP | 0.0005 | n.a. | 76.28 | n.a. | n.a. | 32 | 12 | #5 |
|  |  |  |  |  |  |  |  |  |
| **19 INGROUP TAXA; 48 EXCLUSION SETS** | | | | | | | | |
| **Hybrid taxon: s * l** | | | | | | | | |
| 1726 AFLP | 0.0005 | 0.00001 | 63.25 | 1 (s) | 0 | 61 | 29 | #8 |
| 1285 SNP | 0.0005 | 0.00001 | 76.55 | 1 (s) | 1 | 49 | 22 | #9 |
| 5002 AFLP | 0.0005 | 0.00001 | 79.81 | 0 | 0 | 11 | 5 | #10 |
| 5088 SNP | 0.0005 | 0.00001 | 89.26 | 1 (l) | 0 | 17 | 13 | #11 |
| 5003 AFLP | 0.0005 | 0.000002 | 79.52 | 1 (s) | 0 | 21 | 9 | #12 |
| **Hybrid taxon: (r,q) * k** | | | | | | | | |
| 640 SNP | 0.0005 | 0.000025 | 58.02 | 4 (r,q), 1 (k) | 1 (r,q) | 57 | 30 | #13 |
| 1328 AFLP | 0.0005 | 0.000025 | 64.16 | 1 (r,q) | 0 | 52 | 25 | #14 |
| 1284 SNP | 0.0005 | 0.000025 | 70.07 | 1 (r,q) | 0 | 49 | 24 | #15 |
| 5047 SNP | 0.0005 | 0.000025 | 82.95 | 0 | 0 | 18 | 8 | #16 |
| 964 AFLP | 0.00001 | 0.000025 | 98.28 | 1 (k) | 1 | 4 | 0 | #21 |
| **Hybrid taxon: (r,q) * (l,m,n)** | | | | | | | | |
| 1672 AFLP | 0.0005 | 0.000037 | 63.91 | 0 | 0 | 49 | 16 | #22 |
| 1279 SNP | 0.0005 | 0.000037 | 64.02 | 2 (r,q) | 1 | 68 | 39 | #23 |
| 5160 SNP | 0.0005 | 0.000037 | 85.20 | 0 | 0 | 13 | 7 | #24 |

^1^ Random jackknife sets were composed as follows: a1 q1; a2 p2; c1 u1; c2 hyb1; f2 t1; k1 a2; k2 y2; m1 hyb2; m2 p1; n1 m2; n2 o1; o2 s1; p2 k1; q1 c1; r2 l2; s1 v1; s2 t2; t1 x2; t2 w1; u2 n1; v1 a1; v2 r1; w1 v2; x1 u2; x2 m1; y1 s2; y2 n2; hyb1 x1; hyb2 y1.

Letters represent taxa, numbers 1 and 2 indicate the first and second individual sampled from that taxon.
